# The Body Mass Index, Blood Pressure, and Fasting Blood Glucose in Patients With Methamphetamine Dependence

**DOI:** 10.1097/MD.0000000000003152

**Published:** 2016-03-25

**Authors:** Dezhao Lv, Meijuan Zhang, Xuru Jin, Jiyun Zhao, Bin Han, Hang Su, Jie Zhang, Xiangyang Zhang, Wenwei Ren, Jincai He

**Affiliations:** From the Department of Neurology (DL, JZ, HS, JZ, WR, JH), Department of Clinical Laboratory (MZ), Department of Respiration (XJ), The First Affiliated Hospital of Wenzhou Medical University, Wenzhou, Zhejiang, Department of Nephrology (BH), The First Affiliated Hospital of Jiaxing University, Jiaxing, Beijing HuiLongGuan Hospital (XZ), Peking University, Beijing, China, and Menninger Department of Psychiatry and Behavioral Sciences (XZ), Baylor College of Medicine, Houston, TX.

## Abstract

Methamphetamine (MA) is a prevalently abused psychostimulant in the world. Previously published studies and case reports indicated potential associations between MA and body mass index (BMI) and cardiovascular factors (eg, blood pressure and fasting blood glucose). However, these associations have not been studied clearly. This study aimed to investigate BMI and cardiovascular factors in the MA-dependent patients.

A total of 1019 MA-dependent patients were recruited between February 2, 2008 and March 11, 2013. A case report was used to gather information on sociocharacteristics and drug-dependent history. Meanwhile, a number of 1019 age- and sex-matched controls’ information were collected from the physical examination center. We measured BMI, blood pressure, and fasting blood glucose among the participants.

MA-dependent patients had significantly lower BMI (20.4 ± 0.1 vs 23.9 ± 0.1 kg/m^**2**^, *P* < 0.001), lower fasting blood glucose (5.0 ± 0.01 vs 5.2 ± 0.01 mmol/L, *P* < 0.001) and higher systolic blood pressure (122.1 ± 0.4 vs 114.8 ± 0.4 mmHg, *P* < 0.001) compared with the control group after adjustment of possible confounders. Additional, we only found the duration of MA use was independently associated with BMI (B = −0.08, *P* = 0.04).

This study demonstrated that MA dependence was associated with BMI and cardiovascular factors. In addition, we found a negative association between duration of MA use and BMI.

## INTRODUCTION

Methamphetamine (MA), a popular psychostimulant, has aroused a major public health concern in the world, with some researches reporting that MA could lead to cardiovascular pathology,^1^ hepatic pathology,^2^ mood disorder,^3^ and neurological impairment.^4^ In addition, the long-term MA use leads to poor physical health and malnutrition among the patients.^5,6^

In spite of the possible danger caused by MA, the cardiovascular influences of this drug have not been well clarified. The influence of MA on blood glucose has been reported by most animal experiment. In animal experiment, temporal increase in blood glucose has been observed,^7,8^ which is opposite to a study suggesting that MA administration tends to have hypoglycemia.^9^ On the one hand, a double-blind study has demonstrated that MA could induce severe hypertension, although a study has indicated that hypotension is caused by MA.^10,11^ On the other hand, with body mass index (BMI) defined as the most regularly used and generally accepted method to evaluate the medical risk, there are some studies measuring BMI in MA-dependent patients; however, the results were conflicted.^12,13^ Therefore, the influence of MA on BMI and cardiovascular factors is poorly documented to date.

Given the prevalent use/abuse and the serious cardiovascular effect of MA, an enhanced understanding of cardiovascular responses is needed. It is surprised that little research has investigated cardiovascular responses among MA-dependent patients compared with the healthy population. In our present study, the first purpose was to compare the BMI and cardiovascular factors between a larger Chinese population with MA dependence and healthy controls. Furthermore, we aimed to examine the influence of potential-risk clinical features on BMI and cardiovascular factors among the MA-dependent patients.

## METHODS

### Subjects and Setting

The total of 1019 studied individuals were selected from Sanyang Detoxification Institute between February 2, 2008 and March 11, 2013. All individuals met the following inclusion criteria: age 18 years or above; were only using MA; had a positive result on the urine test for MA at admission; satisfied DSM-IV criteria for MA dependence; have been withdrawal for 1 to 7 days; and signed informed consent. The individuals were excluded if they had dependence on other drugs and had any seriously illness that needed pharmacological treatment.

A total of 1019 healthy age- and sex- matched individuals selected from the regular medical examination at The First Affiliated Hospital of Wenzhou Medical University served as control group, if they had no illegal dependence and serious diseases. All healthy individuals were selected at the same period with the studied ones.

The study was approved by the Human Research and Ethics Committee of Wenzhou Medical University. Written informed consents were signed from all participants.

### Measures

A case report form that included sociodemographic characteristics, age at onset, duration of MA use, routes of drug administration, and daily dose was conducted by the inpatients at admission. The control groups’ sociodemographic data were collected from the physical examination center at The First Affiliated Hospital of Wenzhou Medical University.

### Assessment of BMI, Blood Pressure, and Fasting Blood Glucose

The participants’ height and weight were measured at admission. The BMI was counted as weight divided by the square of height (kg/m^2^). The right arm blood pressure of seated participants was obtained under resting condition. Fasting blood glucose was measured on the second day of admission. All data were recorded by trained operators who blind to the research design.

### Statistical Analysis

We compared the sociodemographic variables, BMI, and cardiovascular factors between MA-dependent patients and healthy controls using analysis of variance (ANOVA), Mann–Whitney *U* test, and *χ*^2^ test as appropriate. When results were significant, we added the possible confounders to the analysis model as covariates to test the effect of these variables. We used the Stepwise multiple regression analysis to explore the effect of clinical features on BMI and cardiovascular factors among the MA-dependent patients. All data were analyzed by SPSS 19.0 (SPSS Inc, Chicago, IL). *P* < 0.05 was considered to be statistically significant.

## RESULTS

### Characteristics of MA-dependent Patients and Healthy Controls

The characteristics of MA-dependent patients and healthy controls are presented in Table 1. There was no significant difference in age and sex between MA-dependent patients and healthy controls (both *P* > 0.5). Among the patients, they had a mean age at onset of MA use of 27.0 ± 7.5 years (range 11–51years). The average duration of MA use was 52.1 ± 58.6 months (range 1–228 months). The routes of MA administration were smoking (91.6%), intranasal administration (5.9%), oral (0.9%), intravenous injection (0.9%), and others (0.7%). The mean dose of MA use was 0.13 ± 0.13 g/day (range 0.05–3.0 g/day).

**TABLE 1:**
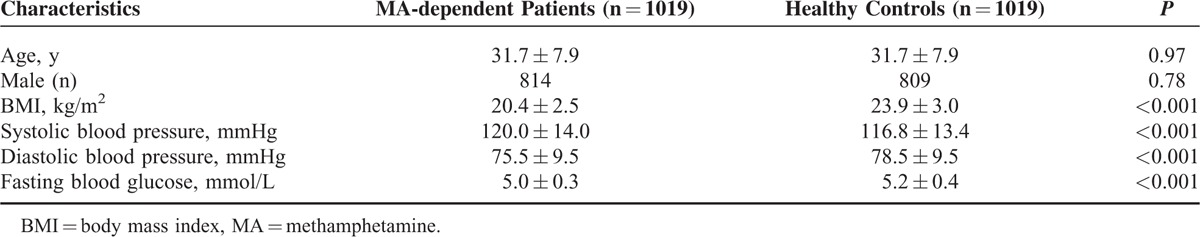
Characteristics of MA-dependent Patients and Healthy Controls

### BMI and Cardiovascular Factors of MA Dependence and Controls

In unadjusted analysis (Table 1), compared with healthy controls, patients had significantly lower BMI (23.9 ± 3.0 vs 20.4 ± 2.5 kg/m^**2**^, *P* < 0.001), fasting blood glucose (5.2 ± 0.4 vs 5.0 ± 0.3 mmol/L, *P* < 0.001), and diastolic blood pressure (78.5 ± 9.5 vs 75.5 ± 9.5 mmHg, *P* < 0.001), whereas systolic blood pressure was significantly higher among MA-dependent patients (116.8 ± 13.4 vs 120.0 ± 14.0 mmHg, *P* < 0.001).

In adjusted analysis (Table 2), there was still significant difference in BMI after age and sex adjustment (*P* < 0.001). However, with the effects of BMI, sex and age adding to the ANOVA as covariates, the significant difference in fasting blood glucose and systolic blood pressure were still existent, except for diastolic blood pressure (*P* < 0.001, *P* < 0.001, *P* = 0.18, respectively).

**TABLE 2:**
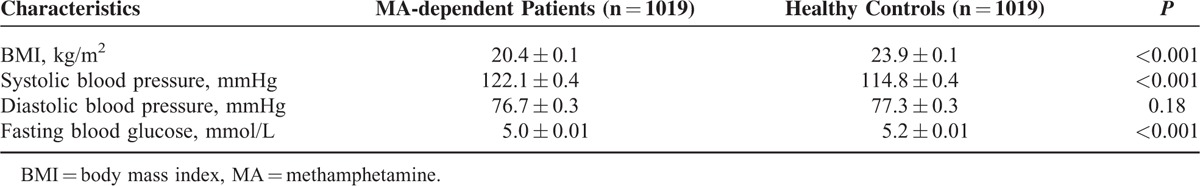
Adjusted Levels of BMI and Cardiovascular Factors Among the Participants

### BMI, Cardiovascular Factors, and Clinical Features Among the MA-dependent Patients

Among the patients, with BMI, systolic blood pressure and fasting blood glucose separately used as a dependent variable and sex, age, the duration of MA use, age at onset, routes of MA administration, and daily dose used as independent variables in the stepwise multiple regression analysis, we only found that the duration of MA use (B = −0.08, *P* = 0.04) was independently related to BMI. However, information on MA-dependent history such as the duration of MA use, age at onset, and daily dose of MA use was not found to be related to systolic blood pressure and glucose (all *P* > 0.05).

## DISCUSSION

In our study, we examined the influence of MA on the BMI and cardiovascular factors in a greater Chinese population. We found that MA-dependent patients had significantly increased systolic blood pressure, decreased BMI and fasting blood glucose than controls. In addition, we found that the duration of MA use was negatively related to BMI. Our results may have vital application in protecting MA-dependent patients from MA complication.

Our study showed that MA caused the expected decrease in BMI as compared with controls, which was in line with previous studies. For example, a study conducted by Suriyaprom et al^12^ suggested that BMIs were lower among MA-dependent patients than controls. Another study performed by Bluml et al^14^ demonstrated that MA may contribute to lower BMI, particularly among adult males. Additionally, the studies performed by Quach et al and Forrester et al showed that drug users had lower BMI than non-drug users among the HIV-infected individuals.^15,16^ Contrary to the results, Barry et al^13^ found that there was no relationship between BMI and drug use disorder in either sex. However, after dropping out nicotine dependence and alcohol use disorders covariates, they found a significant inverse relationship between obesity and past-year illicit drug use disorder in men.

Additionally, according to stepwise multiple regression, we found that the duration of MA use increased a risk of lower BMI. A possible explanation is the long-term influence of MA on BMI. It has been described that dopamine transporter reduction occurred among the MA abusers, with the lower dopamine positively related to the duration of MA use.^4,17^ Furthermore, dopamine has been positively associated with food motivation, suggesting that MA may reduce BMI though dopamine transporter impairment.^18^ However, MA is well known as a powerful liberator and reuptake inhibitor of norepinephrine.^19^ The mechanism of relationship between MA and BMI may be associated to the involvement of norepinephrine. There are many studies prompting a negative relationship between norepinephrine and BMI. For example, a research has revealed that the secretion of norepinephrine in lean women was higher than that in obese ones.^20^ Additionally, the reduction of norepinephrine transporter has been reported in obese individuals.^21^ Therefore, MA may modulate BMI through its stimulating influence on norepinephrine, which may be negatively associated with BMI. Taken together, our study suggested that MA may play an important part in the development of BMI, indicating that the longer duration of MA use was, the less BMI become.

Furthermore, we found that MA dependence was associated with increased blood pressure. Two previous researches are consistent with our findings. For example, a study conducted by Pike et al^22^ showed that elevated systolic blood pressure was positively associated with MA dose. Another double-blind study performed by Stoops et al^11^ suggested that MA produced physiological influence (eg, improved systolic blood pressure, elevated ratings of good effects). Additionally, an animal experiment revealed that elevated blood pressure was attributed to injection of MA administration in rats, which may be correlated to catecholamine liberation.^23^ In contrast to our findings, Burchell et al^10^ reported that hypotension was found in MA-dependent patients during medical treatment. However, the patients were admitted to the hospital with serious metabolic acidosis. A recent study performed by Celotto et al^24^ found that acute metabolic acidosis caused the reduction in blood pressure. In our study, patients who have seriously disease were excluded. Therefore, the discrepancy may be explained by difference in inclusion criteria.

In addition, our study showed that fasting blood glucose in MA-dependent patients was lower than controls, which resembles with previous study. For example, a study reported that the hypoglycemic influence of MA was noted in the mouse. Additionally, this study also demonstrated MA-induced insulin secretion through its direct influence on the pancreas.^9^ Contrary to above results, studies have indicated that MA only increased glucose levels at short time following MA administration in rats.^7,8^ The discrepancy between our study and previous studies demonstrating blood pressure severely increased after MA administration may be explained by the tolerance that develops with MA administration over several days.^25^

There are some limitations in our study. First, we cannot make a conclusion about a casual relationship between MA dependence and BMI and cardiovascular factors because of the cross-sectional design. Second, the information about MA dependence stemmed from self-report of patients, which may lead to deviation with the actual situation. Third, measurement error and observer bias were present in our research as a result of retrospective study. Fourth, we did not examine dietary intake of participants, which may have a biased influence on the results. However, a previous study indicated that the lower BMI of drug abusers could not account by dietary intake.^26^ In contrast with this result, a genetic study demonstrated that appetite regulation genes may be significant markers of risk of developing obesity.^27^ Further studies should be conducted to clarify the relationship between dietary intake and BMI among the MA-dependent patients.

In summary, our study indicated that MA-dependent patients had increased systolic blood pressure, reduced BMI, and fasting blood glucose. Additionally, the duration of MA use was negatively related to BMI. Further studies investigating the causal relationship between MA dependence and BMI and cardiovascular factors should be carried out to develop better treatments to prevent MA complication.
